# The Influence of Fine Particulate Matter and Cold Weather on Emergency Room Interventions for Childhood Asthma

**DOI:** 10.3390/life14050570

**Published:** 2024-04-29

**Authors:** Chih-Chun Hsiao, Chun-Gu Cheng, Zih-Tai Hong, Yu-Hsuan Chen, Chun-An Cheng

**Affiliations:** 1 Department of Nursing, Taoyuan Armed Forces General Hospital, Taoyuan 32549, Taiwan; 2 Department of Emergency Medicine, Taoyuan Armed Forces General Hospital, Taoyuan 32549, Taiwan; 3 Department of Emergency Medicine, Tri-Service General Hospital, National Defense Medical Center, Taipei 11490, Taiwan; 4 Division of Chest Medicine, Department of Internal Medicine, Cheng Hsin General Hospital, Taipei 11220, Taiwan; 5 Department of Neurology, Tri-Service General Hospital, National Defense Medical Center, Taipei 11490, Taiwan

**Keywords:** fine particulate matter, cold air temperature, pediatric asthma emergency room visits

## Abstract

(1) Background: Children are the most vulnerable to pollution due to their decreased stature, heightened respiratory rate, and frequent outdoor engagement. PM_2.5_, nitrogen dioxide (NO_2_), ozone, and cold weather are associated with pediatric asthma. In this study, we investigated the nexus between air pollution, climate factors, and pediatric asthma emergency room visits (ERVs). (2) Method: Pediatric asthma ERV data for healthcare quality from the Taiwanese National Insurance in the Taipei area were obtained from 2015 to 2019. Air pollution and climate factor data were also collected. Poisson regression was employed to determine the relationships with relative risks (RRs). (3) Results: The incidence of pediatric asthma ERVs decreased, with a crude RR of 0.983 (95% CI: 0.98–0.986, *p* < 0.001). Fine particulate matter (PM_2.5_) had an adjusted RR of 1.102 (95% CI: 1.037–1.172, *p* = 0.002) and a 7.7 µg/m^3^ increase, and air temperature had an adjusted RR of 0.813 (95% CI: 0.745–0.887, *p* < 0.001) comparing between the highest and lowest quarter air temperature associated with pediatric asthma ERVs. (4) Conclusions: This inquiry underscores the positive associations of PM_2.5_ and cold weather with pediatric asthma ERVs. The findings could guide the government to establish policies to reduce air pollution and promote children’s health.

## 1. Introduction

Asthma, a chronic respiratory condition, greatly impacts patients’ quality of life. Various factors contribute to asthma exacerbation, including both outdoor and indoor pollutants, allergens, dust mites, and smoking status. Exacerbation symptoms include shortness of breath and chest tightness, with untreated cases leading to increased airflow obstruction. Severe asthma attacks require emergency department intervention for acute treatment [1].

Children, characterized by a small body mass, frequent outdoor activities, heightened exposure, elevated respiration rates, and reduced nasal protection, are more susceptible to the effects of air pollution than adults. This vulnerability underscores the need for increased attention and governmental efforts to promote children’s health. The highest asthma occurrence rate was noted in individuals aged 1–4 years in 2019 globally [2], emphasizing the importance of targeted interventions and awareness campaigns for this age group. In 2016–2018, 35% of asthma emergency room visits (ERVs) in the United States were attributed to pediatric asthma ERVs [3]. Thus, further research and public health initiatives are warranted to address the underlying factors contributing to pediatric asthma exacerbations and to enhance overall asthma management strategies.

There are negative impacts of air pollutant agents on human cardiopulmonary health. The primary risk factor for chronic respiratory diseases is smoking, and particulate matter (PM) pollution is the second most common risk factor [2]. Air pollution, often described as a silent killer, tends to be underestimated despite its large harmful impact. Air pollutants contribute to oxidative injury in the airways, leading to oxidative stress, chronic inflammation, hyperresponsiveness, remodeling, and increased vulnerability to viral infections, exacerbating asthma [4]. After the adjustment of built-up area indicators and socioeconomic indicators, researchers focused on associations with 5-year mean concentrations of nitrogen dioxide (NO_2_), fine particles with an aerodynamic diameter of ≤2.5 µm (PM_2.5_), particles with an aerodynamic diameter of ≤10 µm (PM_10_), and benzene in the city of Brno, Czech Republic [5]. PM_2.5_, PM_10_, and NO_2_ significantly impact pediatric respiratory events, including pneumonia, asthma, bronchitis, and acute pharyngitis, in southern Taiwan [6]. PM_2.5_ and O_3_ were associated with near-fatal/fatal asthma attacks in one study [7]. The meta-analysis found NO_2_, SO_2_ and PM_2.5_ related to asthma exacerbations in high-income cities [8]. Previous research conducted in Taoyuan, Taiwan, revealed that short-term changes in PM_2.5_, ozone, and cold air temperature influenced the duration of asthma-related ERVs. Additionally, PM_2.5_ was identified as a factor related to pediatric asthma ERVs in northern Taiwan [9,10] and exacerbation in Thailand [11]. PM_2.5_, NO_2_, SO_2_, and O_3_ were significantly associated with increased risks of pediatric asthma ERVs in Shanghai, China [12]. An O_3_-8 h concentration above 80 μg/m^3^ contributed to an increased risk of asthma attacks in children [13]. PM was related to motor vehicle emissions and industrial production was related to increasing pediatric asthma visits in USA [14]. Taipei city, which has heavy traffic congestion, has the highest urbanization in Taiwan. Cold weather, in particular, can induce bronchial constriction [15] and childhood respiratory problems. The previous study found that mediating effects of PM_2.5_ increased the severity of chronic obstructive pulmonary disease in cold weather [16]. Thus, investigating the associations between air pollution, climate factors, and pediatric asthma ERVs in Taipei is essential for a comprehensive understanding of the environmental determinants impacting pediatric respiratory health.

The aim of this study was to investigate the potential effects of air pollutants and climate factors on pediatric asthma ERVs in Taipei. Using government-provided open data retrieved from the Taiwan National Insurance Dataset, we aimed to elucidate the potential relationships between asthma exacerbations and childhood asthma exacerbations. The findings may serve as a valuable resource for evidence-based policy development aimed at improving the respiratory health outcomes of the pediatric population in the specified area.

## 2. Materials and Methods

### 2.1. Pediatric Asthma Emergency Room Visits and Air Pollution

**Figure 1:**
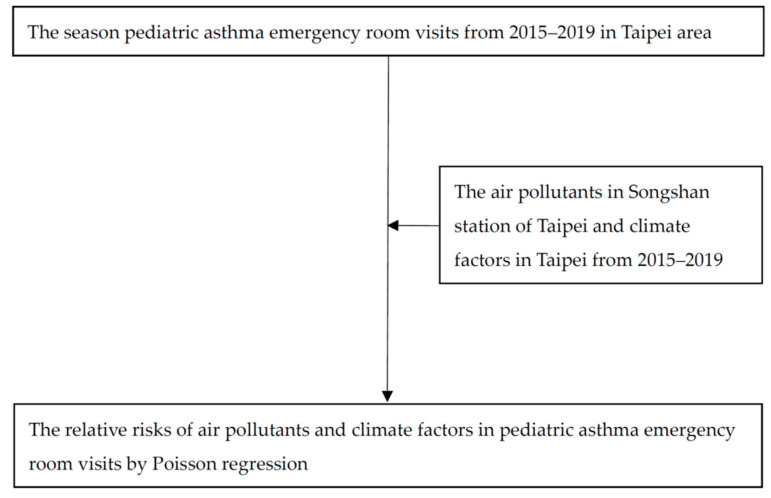
Flowchart of this study.

We utilized healthcare quality reports published by the National Health Insurance Administration every quarter of the year from 2015 to 2019 and used pediatric asthma ERVs as a key indicator of healthcare quality. The numbers of pediatric asthma ERVs and pediatric asthma patients were collected from the Taipei area (Taipei and New Taipei cities) every 3 months. The first quarter was from January to March, the second quarter was from April to June, the third quarter was from July to September, and the fourth quarter was from October to December. Pediatric asthma patients younger than 18 years of age with the ICD10-CM code J45 who had at least four outpatient visits within a year and who used asthma medications were identified from the National Health Insurance Data retrieved from the National Health Insurance Admiration every quarter of year [17]. For the Taipei area, which has a basin topography, we selected the Songshan station in the center of the basin. Air pollutant data were collected from the Songshan station in the center of Taipei, at 25°04′50″ N, 121°58′05″, from the Taiwanese Environment and Weather Administration [18]. The air temperature and relative humidity data were obtained from the Taiwanese Central Weather Administration every month [19]. This study was approved by the Institutional Review Board of Tri-Service General Hospital (protocol code C202405021). The study flowchart is shown in Figure 1. The mean incidence of pediatric asthma ERVs, air pollutant agents and climate factors during the study period are shown in Table 1.

While high correlation coefficients (≥0.8) were noted between each air pollutant, one air pollutant must be excluded. NO_2_ was related to ozone formation via photochemical reactions on hot days. CO_2_ levels below 1000 ppm were considered to indicate no respiratory hazard. The highest daily concentration of CO was 0.6 µg/m^3^, lower than the 4 µg/m^3^ annual average of the WHO air quality guidelines [20] that were not analyzed.

### 2.2. Statistical Analysis

Descriptive statistics, mean pediatric asthma ERVs, air pollutant agents, and climate factors were explored for each quarter. One-way ANOVA tests were conducted to assess the mean values across the four quarters. Poisson regression was used to analyze associations between air pollutant agents, climate factors, and pediatric asthma ERVs by calculating adjusted relative risks (RRs). The PM_2.5_ and air temperature were captured across four quarters. The pediatric ERVs of the highest quarter PM_2.5_ and air temperature were compared with the pediatric ERVs of the lowest quarter in PM_2.5_ and air temperature. Plots were created for the number of pediatric asthma ERVs, air pollutant agents, and meteorological factors each season. Significance was defined at *p* < 0.05, and all the statistical analyses were performed using SPSS version 21.

## 3. Results

**Figure 2:**
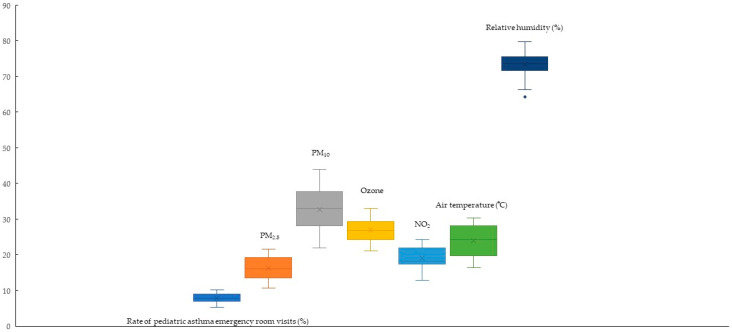
Box plot showing the rate of pediatric asthma emergency room visits, air pollutants and climate factors. Dot means outlier and × means mean values.

The mean number of mean pediatric asthma ERVs was 609 ± 129; the mean rate of pediatric asthma ERVs was 7.9 ± 1.35%; the mean PM_2.5_ was 16.25 ± 3.05 µg/m^3^; the mean O_3_ was 26.91 ± 3.12 ppb; the mean NO_2_ was 19.06 ± 3.04 ppb; the mean air temperature was 23.93 ± 4.53 °C; the mean relative humidity was 73.37 ± 3.76%; the mean CO_2_ was 429.52 ± 6.51 µg/m^3^; the mean methane was 1.81 ± 0.07 µg/m^3^; the mean nonmethane hydrocarbons (NMCHs) was 0.15 ± 0.03 µg/m^3^; the mean total hydrocarbon (THC) was 1.96 ± 0.09 µg/m^3^ during study period. A box plot of the rate of pediatric asthma emergency room visits, air pollutants and climate factors is shown in Figure 2.

The correlation coefficient between PM_2.5_ and CO was 0.9 (*p* < 0.01); the correlation coefficient between PM_2.5_ and NO_2_ was 0.81 (*p* < 0.01); the correlation coefficient between PM_2.5_ and CH4 was 0.81 (*p* < 0.01); the correlation coefficient between PM_2.5_ and THC was 0.89 (*p* < 0.01); the correlation coefficient between CO and NO_2_ was 0.93 (*p* < 0.01); the correlation coefficient between CO and CH_4_ was 0.86 (*p* < 0.01); the correlation coefficient between CO and NMCH was 0.88 (*p* < 0.01); the correlation coefficient between CO and THC was 0.95 (*p* < 0.01); the correlation coefficient between NO_2_ and CH_4_ was 0.89 (*p* < 0.01); the correlation coefficient between NO_2_ and THC was 0.96 (*p* < 0.01). The correlation of air pollutants is shown in Table 1.

**Table 1:** The correlation between air pollutants and climate factors.

	AT	RH	PM_2.5_	O_3_	CO	SO_2_	NO_2_	CH_4_	NMCHs	THC
AT	1	−0.51	−0.44	−0.4	−0.55	0.46	−0.67	−0.69	−0.14	−0.56
*p*		0.02 *	0.05	0.08	0.02 *	0.04 *	<0.01 *	<0.01 *	0.57	0.01 *
RH	−0.51	1	0.07	0.12	0.22	−0.61	0.29	0.1	−0.02	0.2
*p*	0.02 *		0.78	0.62	0.36	<0.01 *	0.22	0.67	0.94	0.4
PM_2.5_	−0.44	0.07	1	0.37	0.9	0.34	0.81	0.81	0.79	0.89
p	0.05	0.78		0.2	<0.01 *	0.14	<0.01 *	<0.01 *	<0.01 *	<0.01 *
O_3_	−0.4	0.12	0.3	1	0.21	−0.04	0.26	0.18	−0.01	0.21
*p*	0.08	0.62	0.2		0.37	0.85	0.26	0.45	0.98	0.37
CO	−0.5	0.22	0.9	0.21	1	0.27	0.93	0.86	0.88	0.95
*p*	0.02 *	0.35	<0.01 *	0.37		0.25	<0.01 *	<0.01 *	<0.01 *	<0.01 *
SO_2_	0.46	−0.61	0.34	−0.04	0.27	1	0.12	0.17	0.49	0.35
*p*	0.04 *	<0.01 *	0.14	0.85	0.25		0.6	0.48	0.03 *	0.13
NO_2_	−0.67	0.29	0.81	0.26	0.93	0.12	1	0.89	0.75	0.96
*p*	<0.01 *	0.22	<0.01 *	0.26	<0.01 *	0.6		<0.01 *	<0.01 *	<0.01 *
CH_4_	−0.69	0.1	0.8	0.18	0.86	0.17	0.89	1	0.67	0.97
*p*	<0.01 *	0.67	<0.01 *	0.45	<0.01 *	0.48	<0.01 *		<0.01 *	<0.01 *
NMCHs	−0.14	−0.02	0.79	−0.01	0.88	0.49	0.75	0.67	1	0.85
*p*	0.576	0.94	<0.01 *	0.98	<0.01 *	0.03 *	<0.01 *	<0.01 *		<0.01 *
THC	−0.56	0.2	0.89	0.21	0.95	0.35	0.96	0.97	0.85	1
*p*	0.01 *	0.4	<0.01 *	0.37	<0.01 *	0.13	<0.01 *	<0.01 *	<0.01 *	

* *p* < 0.05. AT: air temperature; RH: relative humidity; NMCHs: nonmethane hydrocarbons; THC: total hydrocarbon.

**Figure 3:**
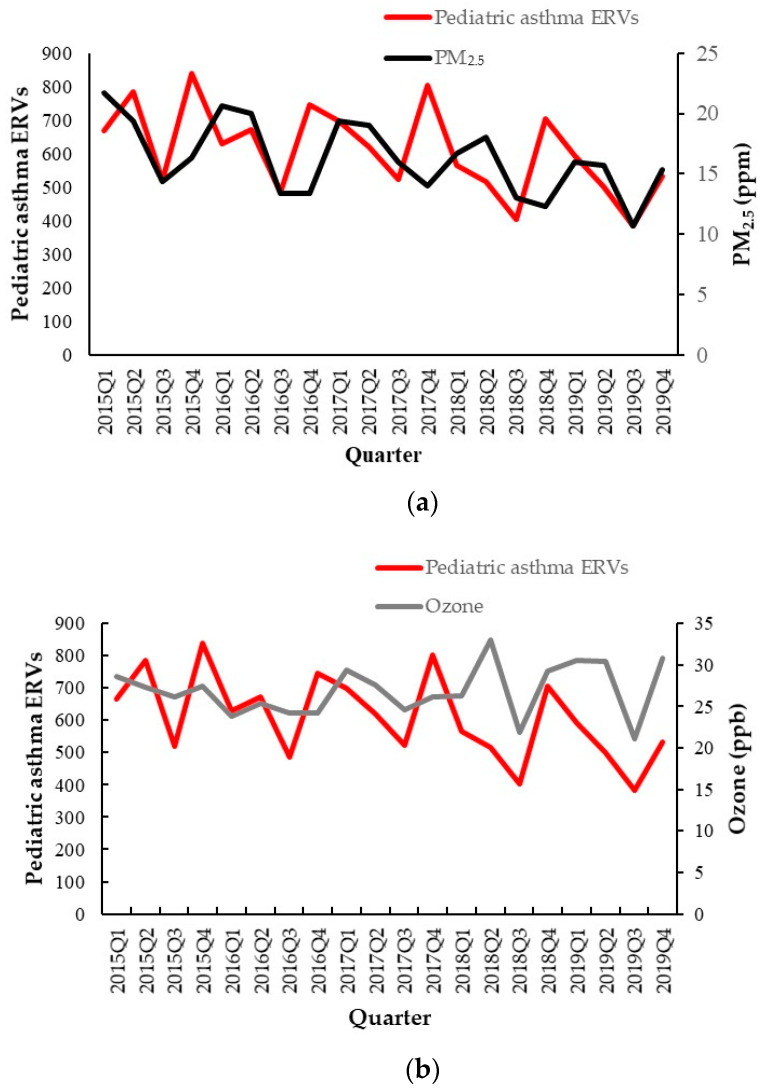

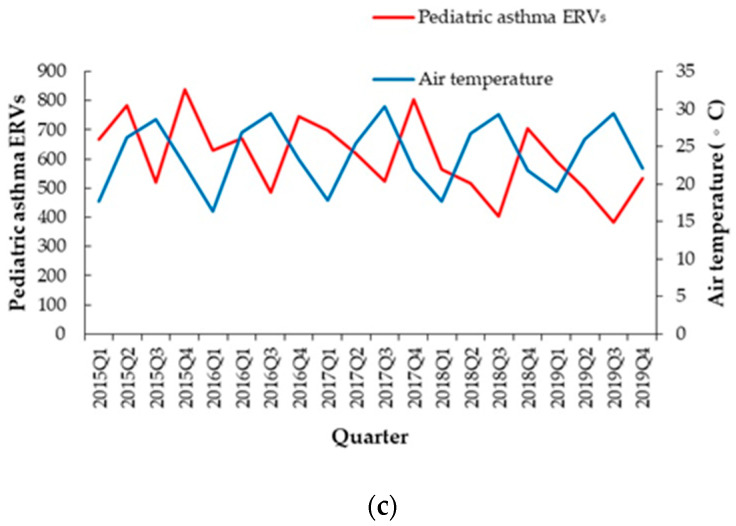
Air pollutants of (**a**) PM_2.5_ and (**b**) ozone; (**c**) climate factors of air temperature and the change in the number of pediatric asthma emergency room visits from 2015 to 2019.

During the study period, the highest mean number of cases of pediatric asthma ERVs was observed in the fourth quarter, at 725 ± 119, while the lowest mean number of cases occurred in the third quarter, at 463 ± 70 (*p* = 0.004). The highest mean PM_2.5_ concentration was 18.87 ± 2.47 µg/m^3^ in the first quarter, and the lowest mean PM_2.5_ concentration was 13.47 ± 1.95 µg/m^3^ in the third quarter (*p* = 0.001). The highest mean O_3_ concentration was 28.74 ± 2.98 ppb in the second quarter, with the lowest mean of 23.59 ± 2.11 ppb in the third quarter (*p* = 0.03). The highest mean air temperature was 29.42 ± 0.59 °C in the third quarter, and the lowest was 17.72 ± 0.93 °C in the first quarter (*p* < 0.001). The highest mean relative humidity was 75.4 ± 2.82% in the first quarter, and the lowest was 70.47 ± 4.26% in the third quarter (*p* = 0.113) (Table 2). The changes in pediatric ERVs, air pollutants and climate factors are shown in Figure 3.

The adjusted RR of PM_2.5_ was 1.102 (95% confidence interval (C.I.): 1.037–1.172, *p* = 0.002) for the highest quarter PM_2.5_ (mean: 20.2 µg/m^3^) compared with the lowest quarter PM_2.5_ (mean: 12.53 µg/m^3^) in pediatric asthma ERVs. The adjusted RR of ambient temperature was 0.813 (95% C.I.: 0.745–0.887, *p* < 0.001) for the highest quarter ambient temperature with a mean of 29.42 °C compared with the lowest quarter ambient temperature with a mean of 17.72 °C. The adjusted RR of ozone was 0.981 (95% C.I.: 0.972–0.99, *p* < 0.001) every 1 ppb increase. The adjusted RR of relative humidity was 0.977 (95% C.I.: 0.99–1.003, *p* = 0.288) every 1% increase (Table 3). In addition, the adjusted RR of NMCHs was 1.013 (95% C.I.: 1.007–1.019, *p* < 0.001) for every 10 ppb increase after adjusting the ozone, relative humidity and air temperature.

The RR of O_3_ was 1.108 (95% C.I.: 1.019–1.205, *p* = 0.016) and air temperature was 0.726 (95% C.I.: 0.56–0.943, *p* = 0.016) during the first quarter. The RR of PM_2.5_ was 1.188 (95% C.I.: 1.114–1.268, *p* < 0.001), O_3_ was 1.053 (95% C.I.: 1.012–1.096, *p* = 0.011) and relative humidity was 1.04 (95% C.I.: 1.018–1.062, *p* < 0.001) during second quarter. The RR of O_3_ was 1.157 (95% C.I.: 1.008–1.329, *p* = 0.038) during the third quarter. The RR of relative humidity was 1.107 (95% C.I.: 1.056–1.16, *p* < 0.001) during fourth quarter (Table 4).

## 4. Discussion

Higher fine particle levels and lower ambient temperatures were associated with greater incidences of pediatric asthma exacerbations, leading to ERVs during the study period. The lower ozone carried a protective effect in pediatric asthma exacerbation. A sustained decline in pediatric asthma ERVs was observed over the study duration. The level of fine particles did not reach the recommended level of less than 5 µg/m^3^ set by the World Health Organization (WHO) [20]. The government should enhance public awareness about the risk effects of air pollution and cold weather on childhood health.

Air pollution reduction policies have been implemented in recent decades, but more efforts have been made to decrease acute childhood asthma attacks [21]. 17.9 percent of ERVs are related to pediatric asthma in the USA [3]. The percentage of pediatric asthma ERVs in Taiwan was lower than that in the USA, decreasing from 10.19% to 6.81% during the study period, with a decreasing trend over time and an RR of 0.98. The potential reason is the availability of qualified asthma care in many clinics and hospitals in Taiwan. The healthcare improvement plan for patients with asthma was implemented by the National Health Insurance Administration several years ago, which has led to a decrease in the incidence of pediatric asthma ERVs in Taiwan [22]. The percentage of patients with mean pediatric asthma ERVs decreased by 28.5% compared with the mean PM_2.5_ decrease of 19.5% from 2015 to 2019. This means that advanced asthma care seems to provide re-education on the prevention of childhood asthma ERVs, reducing their incidence.

PM_2.5_, ozone and NO_2_ cause asthma exacerbations [23]. PM contains inorganic components (carbon, chlorides, nitrates, sulfates, and metals), resulting in potential oxidative stress. The daily number of asthma-related ERVs is related to elemental carbon levels in the summer and winter in adolescents [24]. Local inflammation and persistent free radicals can persist in the ambient environment for 3 weeks [25]. A study revealed that 15% of asthma exacerbations in Europe involved children living close to air pollution sources [26]. PM and NO_2_ are abundant in heavy traffic in urban environments, and PM and NO_x_ are related to pediatric asthma hospitalization [27]. PM_2.5_ can reach the deeper region of the alveoli. PM_2.5_ induced an increase in the levels of kallikreins and the secretion of mucus through barrier activities by epithelial cells [28]. There were primarily transition metals and secondary polar organic compounds with higher free radical stress activity [29]. A previous longitudinal study showed that higher PM_2.5_ concentrations and severe pollen were associated with poorer asthma control [30]. Daily PM_2.5_ exposure was associated with reduced peak expiratory flow and increased symptoms of breathing difficulty [31]. A 10 μg/m^3^ increase in the daily concentration of PM_2.5_ was associated with an increase in pediatric emergency ERVs for asthma or wheezing, with an OR of 1.013, and upper respiratory infections, with an OR of 1.015 [32]. In addition, higher PM_2.5_ exposure carries the risk of respiratory virus infections in Italy [33] and China [34]. Rhinoviruses are possibly the predominant viruses involved in pediatric asthma exacerbation in Taiwan [35]. These respiratory viruses in black and Latinx children with asthma contributed to the prevalence of pediatric asthma ERVs before the COVID-19 pandemic [36]. Rhinoviruses cause almost eighty-five percent of pediatric asthma exacerbations; they attenuate smooth muscle relaxation in the airway [37]. PM_2.5_ and SO_2_ increased in the cold season on lag day 1 (4.9% and 8.57%, respectively) [38]. Higher quartile PM_2.5_ values of TRAPs has been associated with pediatric asthma ERVs [14]. A large amount of traffic was noted in the Taipei metropolis, and PM_2.5_ was difficult to remove from the basin terrain by wind, which resulted in cumulative toxic exposure for several days. Our study found that 10.2% of pediatric asthma ERVs occurred alongside a 7.7 µg/m^3^ PM_2.5_ increase. A spike in the concentration of PM_2.5_ was noted in a past study in Taoyuan due to the burning of Joss paper in April [9]. Our study revealed similar findings with the burning of Joss paper during the Qingming Festival in April, which resulted in a higher PM_2.5_ concentration in the second quarter every year from 2016 to 2019. In addition, higher PM_2.5_ concentrations are associated with seasonal winds, which carry air pollutants from mainland China in colder weather every year.

The mortality and morbidity of asthma and chronic obstructive pulmonary disease are associated with the ambient environment in metropolitan areas in Taiwan [39]. The lower air temperature mediated with higher PM_2.5_ increased the severity of chronic obstructive pulmonary disease in cold weather [16]. A study revealed that short-term cold weather exposure (25% lower in a quarter) related to asthma ERVs at all ages was lower than that related to the mean ambient temperature, with an RR of 1.21 and a 1-day lag [9]. The minimum air temperature in the cold season causes 2.26% of asthma cases and stronger hazard effects for PM_2.5_ and SO_2_ in the cold season with a 1-day lag [38]. A study revealed that higher relative humidity and cold air temperature induced exacerbations of pediatric asthmatic children in Iraq [40]. The cumulative risk of acute URIs increased at an ambient average temperature of 15 °C compared with 33 °C [41]. Our study showed similar findings: every decrease of 1 °C increased the incidence of childhood asthma ERVs by 0.9% after adjustments for other factors. Families and patients must be aware of colder weather to prevent asthma exacerbation during childhood, as this risk increases with decreasing temperature. There were higher air temperatures in the third quarter of every year, causing lower pediatric asthma ERVs. Climate change was related to carbon dioxide, nitrogen oxides (NO_x_) and black carbon [42]. Consequent climate change caused by uncontrolled carbon emissions has been shown to increase the risk of emerging infectious diseases [43]. Global warming has influenced the life cycles of plants, resulting in longer pollen seasons and greater pollen production, causing children to be affected by asthma and allergic rhinitis [44]. Pediatric asthma ERVs are related to PM_2.5_-bound polycyclic aromatic hydrocarbons, with an RR of 1.6 at a one-day lag in Taipei [10]. NMCHs are associated with a 1.3% risk of pediatric asthma ERVs for every 10 ppb increase.

O_3_ induces nonallergic responses [28,45]. This was related to a reduced FEV1 with a one-day lag [31]. O_3_ increased inflammation with nonviral asthma exacerbations in urban areas of the USA [28]. O_3_ was measured only in the summer with photochemical reactions, and the risk increased by 2% with a 1-day lag in warm weather in New York [38]. Asthma exacerbations are related to O_3_ within 8 h of exposure rather than to daily O_3_ [46,47]. O_3_-8 h showed an OR of 1.0503, 95% CI: 1.0277–1.0733 (O_3_ ≥ 100 μg/m^3^) [13]. In addition, ozone is related to good asthma control [22]. O_3_ was negatively related to hospital visits for asthma at concentrations less than 30 ppb in a previous study [48]. Some studies have shown that lower ozone concentrations with low concentrations of ROS are beneficial to cells and have a protective effect against asthma exacerbation [49]. The maximum O_3_ concentration was 33 ppb in Taipei during the study period. Our study showed similar findings with low mean ozone concentrations and fewer pediatric asthma ERVs.

NO_2_ inhaled into alveoli dissolves and produces reactive oxygen species (ROS) and nitrogen substances, inducing oxidative stress and respiratory tract damage [4]. Oxidative stress increased by the generation of ROS and reactive nitrogen species induces chronic inflammation [50]. NO_2_ levels are strongly associated with an increased risk of asthma exacerbation [51]. The daily increase in 10-unit NO_2_ was 7.8% during the warm season in New York [38]. NO_2_ was RR 1.25 at lag 6 days increased pediatric asthma hospitalization Beijing [52]. The NO_2_ cumulative RR of pediatric asthma hospitalization was 1.58 for lag 0–3 days in northern China [53]. Long-term exposure to NO_2_ increases acute upper respiratory tract infections (URIs) [41]. Short-term NO_2_ exposure is not related to pediatric asthma ERVs in Taoyuan. Although there was a 17.5% decrease in the NO_2_ concentration from 2015 to 2019, our study did not examine this due to the greater correlation with PM_2.5_.

Higher relative humidity has been associated with pediatric asthma in Iraq [40]. Elevated relative humidity was initially correlated with pediatric asthma ERVs before adjusting for other factors, but the association became nonsignificant after adjusting for other factors. The higher relative humidity difference mediated with lower PM_2.5_ decreased the severity of chronic obstructive pulmonary disease in warm weather [16]. This observation is attributed to the consistently high mean outside relative humidity exceeding 70% in Taipei compared with 48.03% in Iraq [40], which promoted the growth of dust mites. While families may utilize dehumidifiers to reduce indoor humidity, schools generally lack such equipment. Given that higher relative humidity can stimulate pollen growth and exacerbate childhood asthma [54], we adjusted for relative humidity and found no significant association with pediatric asthma ERVs in the Taipei area. This underscores the complex interplay of environmental factors influencing pediatric asthma outcomes.

Lower air temperature was associated with pediatric asthma ERVs in the first quarter; the potential reasons were bronchial contraction and PM_2.5_ mediation. Higher ozone was associated with pediatric asthma ERVs in the third quarter; the potential reasons were more photochemical action on warm days. Higher relative humidity was associated with pediatric asthma ERVs in the second and fourth quarters; the potential reasons were rainy seasons causing higher relative humidity.

This study revealed a positive effect of PM_2.5_ and cold weather on pediatric asthma ERVs. The air pollution and climate factors related to pediatric asthma outcomes of this study and other studies are shown in Table 5. Despite government efforts to reduce the use of these agents, some asthma-afflicted children experience acute exacerbations requiring urgent emergency care. PM_2.5_ decreased annually (Figure 2). The outlined Sustainable Development Goals encompass objectives such as advancing health and well-being, implementing climate protection measures, and ensuring access to affordable and clean energy [55].

## 5. Limitation

This study has several limitations that warrant consideration. Firstly, the economic status and smoking status of families may introduce confounding variables in the context of childhood asthma. At higher stress exposures, the effects of air pollution are less apparent, indicating potential socioenvironmental interactions [38]. Additionally, the study did not assess indoor air pollutants, which are also related to respiratory health. The incidence of bronchial asthma among younger school-age children is related to indoor air quality in primary schools [57]. High levels of tree pollen were found to be an important risk factor in asthma exacerbations [56]. Furthermore, the heightened risk associated with allergic diseases, exacerbated by both allergens and air pollution, was not explicitly addressed in this investigation. These limitations underscore the need for further research to comprehensively explore the multifaceted factors influencing childhood asthma [58]. Thus, confounding factors need to be studied in the future. Secondly, a notable limitation in our study arises from the government’s collected open data, which were recorded on a quarterly rather than monthly basis. This frequency of data collection may hinder a more detailed examination of the monthly fluctuations in pediatric asthma ERVs and their potential correlation with changes in air pollutants. Each quarter covered 3 months rather than a season. Other studies have adopted a daily mean approach, capturing short-term effects more comprehensively [9,10]. Future research may benefit from more frequent and granular data collection to enhance the temporal resolution of the analysis and provide a more nuanced understanding of the relationship between pediatric asthma ERVs and air pollutant changes. Third, this study was conducted among Chinese participants; the potential variations in other ethnic groups remain unexplored. This study could provide evidence for future studies of other ethnicities. Fourth, our survey focused on the detrimental impacts of air pollutants and ambient temperature on pediatric asthma ERVs, especially in Taipei. Given that regions with greater industrial activity may exhibit distinct effects, further investigations are warranted to examine these potential variations and contribute to a more comprehensive understanding of the nuanced influences of environmental factors on pediatric asthma across different geographic contexts.

## 6. Conclusions

Our study revealed the harmful effect of quarterly PM_2.5_ and cold weather in pediatric asthma ERVs. The pediatric asthma ERVs declined year by year by the government committing to reducing air pollutants and asthma care; however, targeted initiatives to curtail PM_2.5_ levels are still needed to reach the recommended level outlined by the WHO [20]. The NMCHs were found to be part of PM_2.5_ and related to the pediatric respiratory events, which must be reduced. Citizens need to take more public transportation, ride bicycles and walk frequently to reduce TRAP emissions; wearing masks for outside activities and using air purifiers would also reduce PM_2.5_ exposure. Extreme climate conditions are projected to increase the prevalence of pediatric asthma ERVs by 1.9%, coinciding with an 11.7 °C decrease in air temperature. In cold weather, ensuring that children stay warm is crucial for preventing hazards. Lower ozone is associated with fewer pediatric respiratory events; this trend should be continued using aggressive policies. Climate change is intensifying globally and influences human health [59]. Although CO_2_ and CH_4_ were not surveyed for air quality in our study, reductions in CO_2_ and CH_4_ would reduce global warming [60]. Strategies to mitigate climate change often center on clean carbon footprint reduction technologies in England, such as electric vehicles and solar panels [61]. 

City authorities need to enact policies aimed at reducing emissions, monitor the levels of air pollutants, and provide timely warning to at-risk populations. It is valuable for vulnerable asthmatics and sensitive children to receive sufficient warning to avoid certain air pollutants. Through government–citizen collaboration to increase the use of clean energy, thus reducing air pollutant levels and the greenhouse effect, we can contribute to health improvement.

## Figures and Tables

**Table 2:** The mean number of pediatric asthma emergency room visits, air pollutants and climate factors during different quarters.

Quarter	1	2	3	4	*p*
Pediatric asthma ERVs	631 ± 53.83	618 ± 117.09	463 ± 65.93	725 ± 118.87	0.004 *
PM_2.5_	18.87 ± 2.47	18.4 ± 1.69	13.47 ± 1.95	14.27 ± 1.59	0.001 *
PM_10_	38.47 ± 3.57	36.47 ± 3.23	26.27 ± 3.12	29.8 ± 4.77	<0.001 *
NO_2_	22.14 ± 1.78	20.49 ± 2.27	15.57 ± 2.04	18.04 ± 0.7	<0.001 *
O_3_	27.75 ± 2.66	28.74 ± 2.98	23.59 ± 2.11	27.57 ± 2.56	0.03 *
Ambient temperature	17.72 ± 0.93	26.22 ± 0.61	29.42 ± 0.59	22.34 ± 0.56	<0.001 *
Relative humidity	75.4 ± 2.82	72.53 ± 4.03	70.47 ± 4.26	75.07 ± 2.19	0.119

* *p* < 0.05. ERVs: emergency room visits; PM_2.5_: fine particulate matter with an aerodynamic diameter ≤ 2.5 µm; PM_10_: particulate matter with an aerodynamic diameter ≤ 10 µm; NO_2_: nitrogen dioxide; O_3_: ozone.

**Table 3:** The relative ratios of air pollutants and climate factors.

	Relative Ratio	*p*	Adjusted Relative Ratio	*p*
PM_2.5_ highest quarter	1.162 (95% C.I.: 1.105–1.221)	<0.001 *	1.102 (95% C.I.: 1.037–1.172)	0.002 *
PM_2.5_ middle quarter	1.051 (95% C.I.: 1.005–1.1)	0.03 *	1.037 (95% C.I.: 0.982–1.095)	0.191
PM_2.5_ lowest quarter	reference		reference	
Ozone	1.006 (1–1.012)	0.049 *	0.981 (95% C.I.: 0.972–0.99)	<0.001 *
Air temperature highest quarter	0.844 (95% C.I.: 0.8–0.89)	<0.001 *	0.813 (95% C.I.: 0.745–0.887)	<0.001 *
Air temperature middle quarter	1.064 (95% C.I.: 1.02–1.11)	0.004	1.1 (95% C.I.: 1.049–1.153)	<0.001 *
Air temperature lowest quarter	reference		reference	
Relative humidity	1.011 (95% C.I.: 1.005–1.016)	<0.001 *	0.977 (95% C.I.: 0.99–1.003)	0.288

* *p* < 0.05.

**Table 4:** The relative ratios of air pollutants and climate factors across different quarters.

	First Quarter	*p*	Second Quarter	*p*	Third Quarter	*p*	Fourth Quarter	*p*
PM_2.5_	0.98 (95% C.I.: 0.939–1.023)	0.356	1.188 (95% C.I.: 1.114–1.268)	<0.001 *	0.896 (95% C.I.: 0.727–1.103)	0.3	0.893 (95% C.I.: 0.833–0.957)	0.001 *
O_3_	1.108 (95% C.I.: 1.019–1.205)	0.016 *	1.053 (95% C.I.: 1.012–1.096)	0.011 *	1.157 (95% C.I.: 1.008–1.329)	0.038 *	0.941 (95% C.I.: 0.92–0.963)	<0.001 *
AT	0.726 (95% C.I.: 0.56–0.943)	0.016 *	0.952 (95% C.I.: 0.885–1.023)	0.181	1.03 (95% C.I.: 0.88–1.205)	0.714	0.937 (95% C.I.: 0.849–1.034)	0.195
RH	1 (95% C.I.: 0.974–1.027)	0.983	1.04 (95% C.I.: 1.018–1.062)	<0.001 *	0.96 (95% C.I.: 0.897–1.028)	0.241	1.107 (95% C.I.: 1.056–1.16)	<0.001 *

* *p* < 0.05; AT: air temperature; RH: relative humidity.

**Table 5:** Air pollutants and climate factors related to pediatric asthma outcomes.

Reference	Finding	Outcome	Place	Year
Present study	PM_2.5_: RR of 1.102 (95% C.I.: 1.037–1.1722) Air temperature: RR of 0.813 (95% CI: 0.745–0.887)	Pediatric asthma emergency room visits	Taipei, Taiwan	2015–2019
[8]	NO_2_: OR: 1.04 (95% C.I.: 1.001, 1.081) SO_2_: OR 1.047 (95% C.I.: 1.009, 1.086) PM_2.5_: OR 1.022 (95% C.I.: 1.000, 1.045)	Pediatric asthma exacerbations in high-income cities	Meta-analysis	2000–2016
[9]	10 units PM_2.5_: RR 1.195 (95% C.I.: 1.001–1.426) at a 2-day lag	Pediatric asthma emergency room visits	Taoyuan, Taiwan	2016–2019
[10]	10 units PM_2.5_: RR 1.310 (95% C.I.: 1.069–1.606) 10 units PM_2.5_-PAH: RR 1.576 (95% C.I.: 1.371–1.810) on 1-day lag	Pediatric asthma emergency room visits	Taipei, Taiwan	2012–2015
[11]	10 units PM_2.5_: 0.2 events increasing	Pediatric asthma exacerbation	Bangkok and Chiang Mai, Thailand	2020–2021
[12]	10 units PM_2.5_: RR 1.011 (95% C.I.: 1.002–1.021), 10 units NO_2_: 1.030 (95% C.I.: 1.017–1.043), 10 units SO_2_: 1.106 (95% C.I.: 1.041–1.174), 10 units O_3_: 1.009 (95% C.I.: 1.001, 1.017)	Pediatric asthma ERVs	Shanghai, China	2016–2018
[13]	10 units O_3_-8 h increased 6.33% in ≥100 μg/m^3^ O_3_-8 h 10 units O_3_-8 h increased 2.36% in 80–99 μg/m^3^ O_3_-8 h PM_2.5_: OR: 1.0503 (95% C.I.: 1.0277–1.073) in ≥100 μg/m^3^ O_3_-8 h	Pediatric asthma attacks	Xiamen, China	2016–2019
[14]	Higher quartile TRAPs-PM_2.5_: RR 1.789 (1.517–2.109) Higher quartile TRAPs-NO_2_:1.893 (1.589–2.256)	Pediatric asthma emergency room visits	Cleveland, OH, USA	2009–2010
[31]	O_3_: rate ratio 1.52 (95% C.I.: 1.02–2.27) NO_x_: rate ratio 1.61 (95% C.I.: 1.23–2.11) NO: rate ratio 1.80 (95%C.I.: 1.37–2.35)	Rescue inhaler use	Los Angeles, CA, USA	2019
[38]	Cold season PM_2.5_: 4.90% (95% C.I.: 3.77–6.04) on 1-day lag SO_2_: 8.57% (5.99–11.21) on 1-day lag Warm season NO_2_: 7.86% (95% C.I.: 6.66–9.07) on 1-day lag O_3_: 4.75% (95% C.I.: 3.53–5.97) on 2 days lag Minimum air temperature: 2.26% (95% C.I.: 1.25–3.28) in the cold season	Pediatric asthma emergency room visits	New York, NY, USA	2005–2011
[32]	10 units PM_2.5_: OR 1.013 (95% C.I.: 1.003–1.023)	Pediatric asthma or wheeze	GA, USA	2002–2010
[40]	Relative humidity: correlation coefficients of 0.795 Rain days: 0.890 Wind speed: −0.763 Air temperature: −0.837	Pediatric asthma hospitalization	Basra, Iraq	2014–2016
[51]	NO_2_ correlation coefficient: 0.4619 in Buffalo; −0.543 in Detroit, 0.1924–0.3113 Phoenix, 0.2244 in Tucso	Pediatric asthma hospitalization	Buffalo, NY, Detroit, MI, Phoenix, AZ and Tucson, AZ, USA	2009–2011
[52]	NO_2_: RR 1.25 (95% C.I.: 1.06–1.48) at lag06 SO_2_: RR 1.17 (95% C.I.: 1.05–1.31) at lag05	Pediatric asthma hospitalization,	Beijing, China	2013–2016
[53]	NO_2_: cumulative effects 1.580 (95% C.I.: 1.315–1.899, lag 0–3 days	Pediatric asthma hospitalization	Hefei, China	2015–2016
[56]	O_3_: rate ratio 1.05 (95% C.I.: 1.04–1.06) PM_2.5_: rate ratio 1.03 (95% C.I.: 1.02–1.04) The 5-day average values of tree and weed pollen: rate ratio 1.23 (95% C.I.: 1.21–1.25)	Pediatric asthma emergency room visits	NJ, USA	2004–2007

RR: relative risk; OR: odds ratio; C.I.: confidence interval.

## Data Availability

The datasets used in the current study are available from the corresponding authors.
